# Integrating Noninvasive Radiofrequency Monitoring Technology Into Clinical Decision‐Making

**DOI:** 10.1002/alz.090473

**Published:** 2025-01-09

**Authors:** Britni A Bolstad, Brian B Johnson

**Affiliations:** ^1^ WellBe Senior Medical, Atlanta, GA USA; ^2^ University of Minnesota, Minneapolis, MN USA

## Abstract

**Background:**

Across a broad range of physical and cognitive outcomes in gerontology and geriatric practice, it is fundamentally important to establish systems for clinical decision‐making. Current technological solutions include sensors for monitoring movement activity, however many of these methods are complex, invasive, and dependent on factors such as compliance, context, and idyllic conditions. An alternative approach to monitoring is through continuously active, noninvasive radiofrequency (NRF) sensors. Our hypothesis is that NRF sensors can be used to detect clinically relevant changes and support clinical decision‐making because they provide a more accurate and reliable method for monitoring movement activity than camera‐based systems and wearable sensors, thus the identification and progression of physical and cognitive decline could be measured through movement data and enhance the care management of older adults.

**Method:**

NRF sensors are highly suited to optimizing supportive actions by clinicians and healthcare organizations. However, despite the interest in NRF systems, it is mostly absent from clinical context aside from research initiatives. Commercial technologies and technologies developed in research initiatives are often developed without a proper understanding of the specific needs of older adults or what clinicians consider useful. Our approach is to use NRF sensors and an algorithm library to identify changes in movement activity and inform clinical decision‐making thereby facilitating care management based on individual needs.

**Result:**

Initial lab tests at the University of Minnesota showed the NRF sensor to be effective for monitoring falls in real‐time, movement activity, and resting states. These results suggest that NRF sensors could be used to measure clinically relevant changes among the older adult population.

**Conclusion:**

NRF sensors may provide clinicians with pertinent data that would otherwise be inaccessible and help them to develop an individualized plan of care. With movement data, clinicians could further assess risk factors, monitor for adverse events, and detect physical or cognitive decline to improve care management. NRF monitoring technology is conceptualized as a means of better fulfilling the needs of older adults. Further research questions seek to evaluate the impact of using NRF sensors for quantitative monitoring, particularly within healthcare organizations.

